# Ischemic Preconditioning: Modulating Pain Sensitivity and Exercise Performance

**DOI:** 10.3389/fphys.2021.696488

**Published:** 2021-06-22

**Authors:** Joshua T. Slysz, Jamie F. Burr

**Affiliations:** Department of Human Health and Nutritional Sciences, University of Guelph, Guelph, ON, Canada

**Keywords:** athletic performance, ischemia, analgesia, endogenous opioids, antinociception

## Abstract

**Purpose** The purpose of this study was to examine whether an individual’s IPC-mediated change in cold pain sensitivity is associated with the same individual’s IPC-mediated change in exercise performance.

**Methods** Thirteen individuals (8 males; 5 females, 27 ± 7 years, 55 ± 5 ml.kgs^–1^.min^–1^) underwent two separate cold-water immersion tests: with preceding IPC treatment and without. In addition, each participant undertook two separate 5-km cycling time trials: with preceding IPC treatment and without. Pearson correlation coefficients were used to assess the relationship between an individual’s change in cold-water pain sensitivity following IPC with their change in 5-km time trial performance following IPC.

**Results** During the cold-water immersion test, pain intensity increased over time (*p* < 0.001) but did not change with IPC (*p* = 0.96). However, IPC significantly reduced the total time spent under pain (−9 ± 7 s; *p* = 0.001) during the cold-water immersion test. No relationship was found between an individual’s change in time under pain (*r* = −0.2, *p* = 0.6) or pain intensity (*r* = −0.3, *p* = 0.3) following IPC and their change in performance following IPC.

**Conclusion** These findings suggest that IPC can modulate sensitivity to a painful stimulus, but this altered sensitivity does not explain the ergogenic efficacy of IPC on 5-km cycling performance.

## Introduction

Ischemic preconditioning (IPC) traditionally involves the exposure of brief periods of circulatory occlusion and reperfusion to a limb that activates protective mechanisms against ischemic-reperfusion injury in local tissues (Kharbanda et al., 2002; Addison et al., 2003). The identity of the protective triggers, and mechanisms by which the trigger is conveyed from the IPC stimulus to the tissue, remain incompletely understood; however, previous evidence has implicated an important role of opioids as an endogenous substance released by preconditioned tissue (Tomai et al., 1999; Dickson et al., 2002; Patel et al., 2002). It is known that opioid release and activation of opioid receptors at the peripheral, spinal, or supraspinal level can modulate ascending pain information (Benarroch, 2012). As such, the local release of endogenous opioids via IPC may act to modulate pain sensitivity. In the clinical setting wherein patients have a pre-existing baseline pain, IPC administration prior to surgery can reduce patient-reported post-operative pain (Memtsoudis et al., 2010; Pereira et al., 2016).

IPC applied prior to exercise has been shown to improve high-intensity exercise performance, both when brief bouts of circulatory occlusion are applied to locomotive (De Groot et al., 2010; Crisafulli et al., 2011) or remote limbs (Jean-St-Michel et al., 2011; Barbosa et al., 2015). The mechanisms underlying these exercise improvements are unknown (Incognito et al., 2016). It has been shown that levels of endogenous opioids influence effort perception during an exercise task and resulting exercise performance (Sgherza et al., 2002). As such, endogenous opioid release with IPC (Tomai et al., 1999; Dickson et al., 2002; Patel et al., 2002) may reduce the perception of effort during a high-intensity exercise task, allowing for an increased effort and performance. There appears to be large between-subject variability in the exercise performance response to IPC, possibly owing to the existence of responders and non-responders to IPC treatment (Incognito et al., 2016). It is possible that an individual’s exercise performance response to IPC will be explained by the ability of IPC to modulate one’s own sensitivity to pain or discomfort. Therefore, the purpose of this study was to examine whether IPC-mediated changes in cold pain sensitivity are associated with the same individuals IPC-mediated change in exercise performance. We hypothesized that a greater IPC-mediated reduction in cold-water pain sensitivity would be associated with a greater IPC-mediated improvement in 5-km cycling time trial performance.

## Materials and Methods

### Subjects

Thirteen aerobically trained individuals (8 males: 27 ± 7 years, 178 ± 4 cm, 77 ± 7 kg, 55 ± 5 ml.kg^–1^.min^–1^, and 5 females: 26 ± 6 years, 168 ± 7 cm, 63 ± 12 kg, 55 ± 7 ml.kg^–1^.min^–1^) volunteered to participate in this study, which employed a randomized cross-over design. Participants were non-smokers, with no medical history of chronic disease and were safe to exercise as confirmed through completion of a PARQ+ screening questionnaire (Bredin et al., 2013). After being advised of the purpose and potential risks of the study, participants provided written informed consent in accordance with the guidelines of the institutional human research ethics board who approved the experimental protocol and procedures of this study.

### Familiarization

During a performance familiarization visit, participants completed a maximal oxygen consumption test ($\dot{V}$O_2_max) on a cycle ergometer (Velotron Inc., Seattle, United States) to evaluate aerobic fitness. The test began with a resistance of 100 W and increased continuously (1 watt every 3 s) until attainment of $\dot{V}$O_2_max. Attainment of true physiological max was confirmed for all participants by the presentation of a plateau in $\dot{V}$O_2_ (increase in ≤ 50 mL/min at $\dot{V}$O_2_ peak and the subsequent data points), and respiratory exchange ratio (RER) ≥1.15 (Astorino et al., 2000). Expired gases were measured via indirect calorimetry using a face mask and optical turbine connected to a gas analyzer with a sampling line (Cosmed Quark CPET, Rome, Italy). The maximal values were recorded as the highest reading that occurred after the data was smoothed using a rolling 30 s average. Three to five days following the $\dot{V}$O_2_ max test, participants completed a familiarization 5-km TT to acquaint themselves with the gearing functions of the cycle ergometer and pacing strategies for the 5-km distance. Finish time was not recorded. Immediately following the familiarization 5-km TT, each participant briefly submerged his or her hand into cold water for a total of 10 s to acquaint themselves with the cold pain stimulus. A complete cold-water immersion test was not completed for familiarization purposes to avoid over-familiarization and undesirable adaptation.

### Experimental Design

Each participant performed two separate cold-water immersion tests: (i) with preceding IPC administration (CWI_IPC_), (ii) without preceding IPC administration (CWI_CON_). These tests were completed on separate days with at least 3 days between. The order of the two tests was randomized by the flip of a coin. Participants refrained from alcohol, caffeine, and intensive physical exercise for a least 24 h prior to both CWI tests. Pain induced by the submergence of the hand in cold water has been used in investigating a wide range of pain management techniques (Mitchell et al., 2004) and has an excellent reliability and validity (Edens and Gil, 1995). Since previous evidence has implicated an important role of opioids as the endogenous substance that is released by the preconditioned tissues (Tomai et al., 1999; Dickson et al., 2002; Patel et al., 2002), cold was chosen as a stimulator for pain as it has previously been shown that activation of μ-opioid receptors at the peripheral level can modulate cold pain information (Staahl et al., 2009).

**FIGURE 1:**
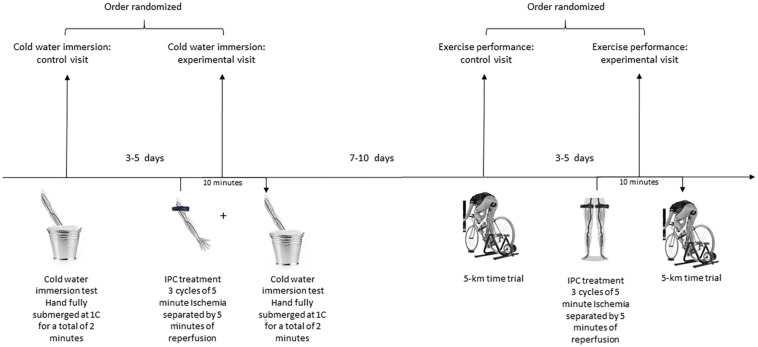
Schematic Overview of the experimental design. Each participant underwent two separate cold-water immersion tests: with preceding unilateral IPC administration to the left arm (experimental visit) and without (control visit). Each participant also underwent two 5-km time trials: with preceding bilateral IPC administration to the legs (experimental visit) and without (control visit).

Each participant also underwent one experimental IPC and one control 5-km cycling time trial. These trials were completed on separate days with a minimum of 3 days between trials to allow sufficient recovery time. The order of the two tests was randomized by the flip of a coin. Prior to both trials, participants completed a 15 min warm-up at a power output of 100 W on a cycle ergometer. Participants refrained from alcohol, caffeine, and intensive physical exercise for a least 24 h prior to performance testing. The experimental design is summarized in Figure 1.

### Cold-Water Immersion: Test Protocol

With the participant seated, the left hand was immersed to above the wrist in a bucket of water at 1 degree Celsius for a total of 2 min. Each participant was instructed to say “pain” when the cold stimulus first became painful. At the end of the 2 min, participants were asked to remove their hand from the cold water and instructed to say “no pain” when the perception of pain had subsided. The time (s) from when the participant first reported pain to when pain was completely gone after removal from the cold stimulus was used to measure total time under pain. Two minutes was set as an upper limit to ensure all participants could complete the entire test without prematurely removing his/her hand. This allowed for water immersion time to be the same in both tests and total time under pain to be compared. Throughout the 2-min of water immersion, each participant was asked to rate the intensity of pain on a 0–5-point Likert rating scale every 15 s from “no pain” to “excruciating,” as described by the McGill pain questionnaire (Melzack, 1987). Participants were naïve to the expected IPC outcome and no verbal encouragement was given throughout the test.

Throughout both cold-water immersion tests, beat-to-beat blood pressure was continuously measured from a digit photoplethysmography cuff (Human Non-Invasive Blood Pressure (NIBP); ADInstruments-North America, Colorado, United States) applied to the right hand. This measurement was also used to measure heart rate. Systolic blood pressure (SBP), Diastolic blood pressure (DBP), heart rate (HR) were measured for 2 min at baseline (Pre), during CWI (Mid), and 2 min following hand removal from cold water (Post).

### Cold-Water Immersion: IPC Protocol

For CWI_IPC_, an IPC protocol was fully completed 15 min prior to the test. IPC was performed in a seated position using a PTSi automated tourniquet system (Defli Medical Innovations Inc. Vancouver, Canada) with unilateral arterial occlusion of the left arm. The tourniquet cuff was positioned proximally and inflated to a pressure superior to brachial systolic pressure (≥2 mmHg), allowing complete arterial occlusion. This pressure, called the lowest effective occlusion pressure (LOP), can be detected by the Delfi system for each participant by utilizing a pressure transducer to determine the pressure required to cause the arterial pulsation to disappear (Masri et al., 2016). LOP was determined in duplicate and the average of these two values was used to set the pressure for circulatory occlusion. Circulatory occlusion lasted 5 min and was performed three times, each separated by 5 min of reperfusion (Patterson et al., 2019). No participant reported undue pain or discomfort during the arm circulatory occlusion.

### Exercise Performance: 5-km Cycling Time Trial Protocol

All cycling time trials were completed on the same stationary cycle ergometer as the VO_2_max test which uses an electromagnetic resistance. Integrated 3D computer software was used to complete a flat, 5-km race, wherein time to completion (s), average and peak power output (Watts), and average revolutions per minute (RPM) were recorded. The system allowed participants to change gears virtually and select their own resistance/RPM to suit their desired pacing strategy. On a computer monitor participants viewed the image of a rider on course (representing the test subject), but were blinded for elapsed time, speed, power output, RPM and specific gearing level, as these variables were removed from the software’s display interface during each trial. Participants were also blinded to the precise distance covered, however, a general guide using a linear scale and representative marker was available for their reference to allow for pacing their work output toward the known endpoint of 5 km. This distance was chosen based on previous research that suggests the most consistent benefit of IPC is for an improvement in time-trial performance in high-intensity exercise tests approaching aerobic capacity (Incognito et al., 2016). In addition, this distance involves less pacing strategy, and was thus chosen to minimize any pacing variability or motivational influences that occur during a prolonged cycling task. Participants were naïve to the expected IPC outcome and received no verbal encouragement throughout any cycling test to avoid being influenced by the researchers.

### Exercise Performance: IPC Protocol

Prior to the experimental trial, an IPC protocol was fully completed 15 min prior to the start of the trial. IPC was performed in a seated position using bilateral arterial occlusion of the legs. The tourniquet cuffs were positioned proximally on the thighs and inflated to a pressure that was minimally superior to femoral systolic pressure (≥2 mmHg) using the Delfi system as described above. Circulatory occlusion lasted 5 min and was performed three times, each separated by 5 min of reperfusion. No participant reported undue pain or discomfort during the leg circulatory occlusion. Application of cuffs to the legs was chosen to keep the IPC stimulus local to the pain stimulus, as cuff application and cold-water immersion involved the same limb.

### Data Analysis and Statistics

To determine the IPC-changes in cold pain sensitivity, the difference in total time under pain between CWI_CON_ and CWI_IPC_ was compared using a paired sample *t*-test. Differences in the intensity of pain throughout the 2 min of cold-water immersion were compared between CWI_CON_ and CWI_IPC_ using a 2 (group) × 8 (time point) repeated measures ANOVA with *post-hoc* comparisons if appropriate. Hemodynamic parameters were compared at Pre, Mid, and Post, between CWI_CON_ and CWI_IPC_ using a 2 × 3 repeated measures ANOVA with *post-hoc* comparisons if appropriate.

The ergogenic effect of IPC on 5-km cycling time trial performance was assessed by calculating percent change between the control and experimental IPC trial. Pearson correlation coefficients were used to quantify and compare the association of the ergogenic effect of IPC with the pain sensitivity modulation of IPC. Specifically, Pearson correlation coefficients were used to relate the percent change in performance with the change in time under pain (s). In addition, area under the curve (AUC) was calculated from the reported pain intensity Likert scores of CWI_CON_ and CWI_IPC._ The change in pain intensity (AUC) between the two trials was also related to the ergogenic effect of IPC. All Statistical analyses were conducted using SPSS software (version 25; IBM, Chicago, IL, United States), with differences considered to be statistically significant at *P* < 0.05. Data is presented as mean ± SD, unless specified otherwise.

## Results

### Pain Sensitivity Modulation

**FIGURE 2:**
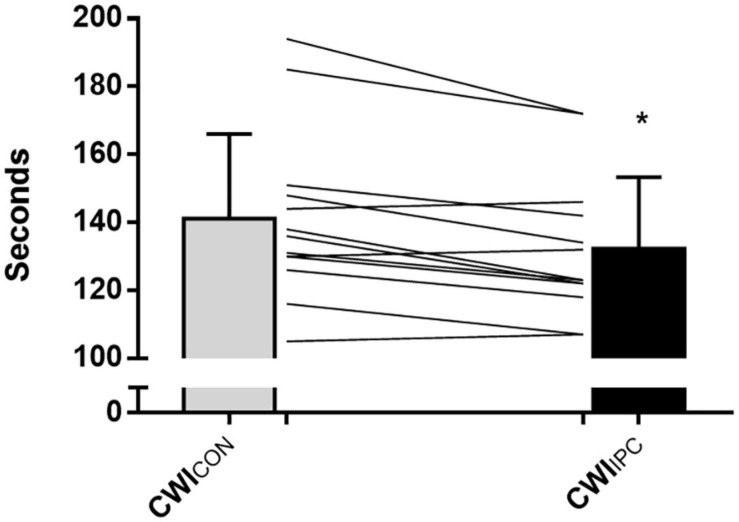
Time (s) under pain during a cold-water immersion test under normal conditions (CWI_CON_) and following IPC treatment (CWI_IPC_). The time under pain represented the total time (s) from when the participant first reported pain after introduction of the cold stimulus to when the pain was completely gone after removal of the cold stimulus. Data is represented as mean ± SD, and * represents a statistically significant difference (*P* < 0.05).

**FIGURE 3:**
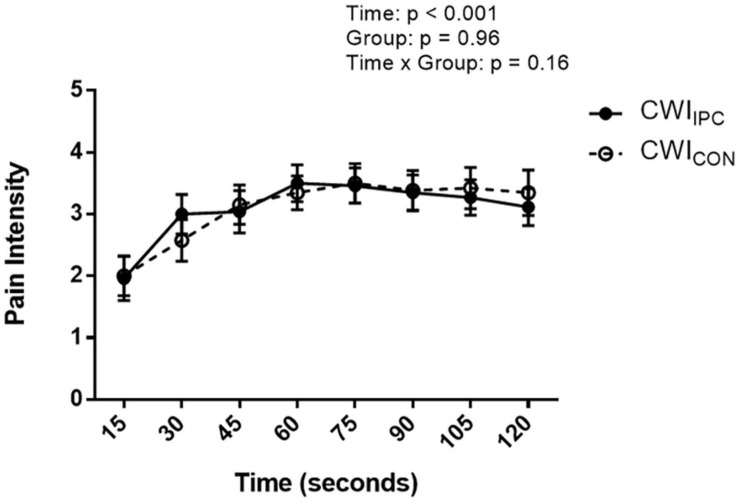
Ratings of pain intensity as reported on a 1–5-point Likert scale every 15 s during a cold-water immersion test in control (CWI_CON_) and experimental IPC (CWI_IPC_) conditions. Data is represented as mean ± SD.

All participants reached the maximum time of 2 min in both CWI_CON_ and CWI_IPC_. There was a significant reduction in time under pain after IPC (132 ± 20 s) compared to control (141 ± 24 s; Cohen’s *d* = 1.3; *p* = 0.001; Figure 2). Pain intensity increased with progressive time in both CWI tests (*p* < 0.001), however, this rate of increase was not altered by IPC (*p* = 0.96), nor did IPC have an effect on pain intensity at any point throughout the test (*p* = 0.16; Figure 3). SBP, DBP, MAP, and HR increased with progressive time in both CWI tests (*p* < 0.05), however, these responses were not different following IPC (*p* > 0.05), nor did IPC influence SBP, DBP, MAP, or HR at any point throughout the test (*p* > 0.05; Table 1).

**TABLE 1:** Hemodynamic parameters measured throughout a cold-water immersion test without (CWI_CON_) and with (CWI_IPC_) preceding IPC administration.

**Variable**		**Pre**	**During**	**Post**	***P*-value**
		**(2 min)**	**(average)**	**(2 min)**	
SBP (mmHg)	CWI_CON_	123 ± 7	148 ± 14	135 ± 15	Group = 0.8
	CWI_IPC_	123 ± 11	148 ± 18	132 ± 14	Time < 0.001
					Interaction = 0.5
DBP (mmHg)	CWI_CON_	66 ± 11	82 ± 11	72 ± 11	Group = 0.9
	CWI_IPC_	65 ± 11	83 ± 12	72 ± 12	Time < 0.001
					Interaction = 0.6
MAP (mmHg)	CWI_CON_	85 ± 10	104 ± 12	93 ± 12	Group = 0.9
	CWI_IPC_	84 ± 11	105 ± 14	92 ± 13	Time < 0.001
					Interaction = 0.6
HR (beat/min)	CWI_CON_	65 ± 12	75 ± 11	65 ± 10	Group = 0.6
	CWI_IPC_	67 ± 8	76 ± 12	66 ± 9	Time < 0.001
					Interaction = 0.9

*Data is represented as mean ± SD.*

### Exercise Performance vs. Pain Sensitivity Modulation

**FIGURE 4:**
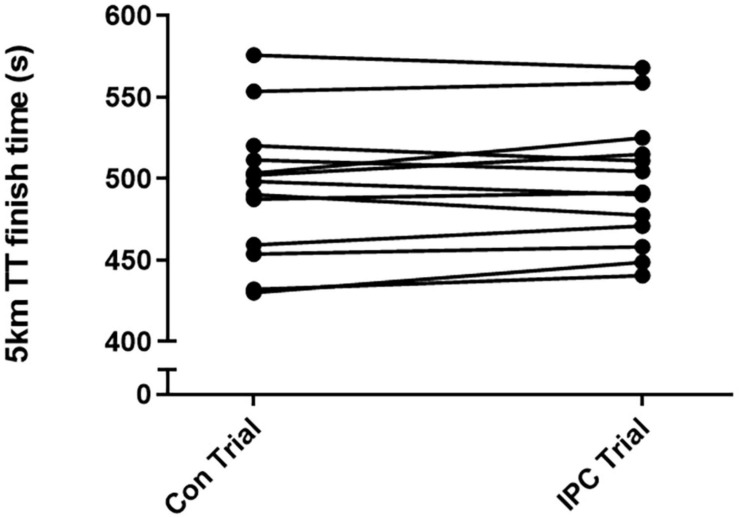
Individual finish times in seconds of the 5-km time trial under normal conditions (Con trial) and following IPC (IPC trial). Of 13 participants, 8 improved exercise performance following IPC while 5 did not.

Mean performance improved with IPC by 0.8 ± 2% over the 5-km TT, however, as a result of large inter-individual differences driving variability, this mean performance improvement did not reach statistical significance (Con Trial: 497 ± 39 s vs. IPC trial: 494 ± 43 s, *p* = 0.3; Figure 4). Of all 13 participants, 8 (62%) improved 5-km TT performance following IPC (Con Trial: 488 ± 42 vs. IPC Trial: 477 ± 42 s, *p* = 0.002), while 5 (38%) did not improve 5-km TT performance following IPC (Con Trial: 510 ± 35 s vs. IPC Trial: 519 ± 34 s, *p* = 0.001).

**FIGURE 5:**
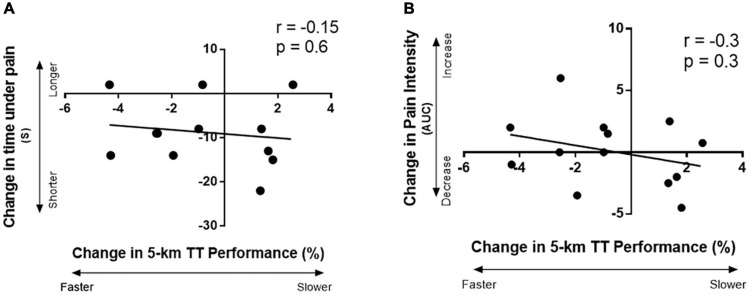
Correlation between the change in 5-km TT performance with IPC with: **(A)** the change in time under pain (s) after IPC (time under pain represented total time from when the participant first reported pain after introduction of the cold stimulus to when the pain was completely gone after removal of the cold stimulus), and **(B)** the change in pain intensity area under the curve with IPC; area under the curve was calculated from the reported pain intensity Likert scores from a cold-water immersion test.

There were no significant associations between the percent change in performance with IPC and the change in time under pain with IPC (*r* = −0.2, *p* = 0.6, Figure 5A), or the change in pain intensity (AUC) with IPC (*r* = −0.3, *p* = 0.3, Figure 5B).

## Discussion

The present study sought to investigate whether an individual’s IPC-induced change in pain sensitivity are associated with the same individual’s IPC-induced change in 5-km cycling time trial performance. The main findings were that (1) IPC administration prior to a cold-water immersion test decreased total time under pain; however, it did not change perception of pain intensity during the cold stimulus, (2) IPC-induced changes in pain sensitivity were not related to IPC-induced changes in 5-km time trial performance. These findings provide evidence that IPC reduces external pain sensitivity; however, an individual’s change in exercise performance after IPC is not explained by the IPC effect on external pain sensitivity.

### IPC Ergogenic Effect vs. IPC Pain Modulation

Certain types of high intensity exercise are perceived as painful. Indeed, reproducible relationships between objective measures of exercise intensity and subjective assessment of leg muscle pain intensity during cycle ergometry have been reported (Cook et al., 1997). Therefore, if IPC can decrease an individual’s perception of this pain, his/her performance response following IPC may be explained by a decrease in muscle pain sensitivity during an intense exercise task, allowing for increased effort and improved performance. However, in the current study, IPC-mediated reductions in pain sensitivity were not related to IPC-mediated improvements in performance. This finding suggests that a reduction in pain sensitivity did not explain an individual’s performance response following IPC. In addition, a reduction in pain sensitivity did not relate to a verified IPC performance responder, providing evidence that reductions in pain sensitivity do not explain the ergogenic effect of IPC.

The aforementioned findings were unexpected given that previous literature demonstrates an improved exercise performance after IPC administration without corresponding improvements in aerobic metabolism (Cook et al., 1997; Crisafulli et al., 2011; Slysz and Burr, 2018). It is still possible that IPC improves exercise performance through perceptual modulation, as the mechanism by which IPC modulates an individual’s perception to cold pain may not equate to how IPC modulates his/her perception of exercise. It is important to note that the pain of a cold-pressor test is driven by a single external stimulus, whereas the discomfort of intense exercise is internal and dependent on multiple signals across organs. Along these lines, it has been alternatively theorized that IPC may improve performance by modulating sensitivity to fatigue rather than pain (Crisafulli et al., 2011; Slysz and Burr, 2018). Future research should continue to investigate the possibility that IPC-induced improvements in exercise performance are related to perceptual changes during the exercise task.

### IPC Pain Modulation

To date, little work has investigated the use of an IPC protocol on reducing sensitivity to a purposely introduced painful stimulus in healthy individuals. Previous literature, however, has investigated the analgesic effect of IPC in a clinical setting, indicating that postoperative pain intensity (Memtsoudis et al., 2010; Pereira et al., 2016) morphine use (Memtsoudis et al., 2010), and mean hospital stay (Pereira et al., 2016) can be reduced in patients who undergo IPC before a surgical procedure. In like manner, the current study found that IPC can modulate pain sensitivity by reducing total time under pain; in contrast, however, absolute pain intensity was not affected. It should be pointed-out that previous clinical research (Memtsoudis et al., 2010; Pereira et al., 2016) evaluated pain intensity in the recovery from a painful stimulus, while the current study evaluated pain intensity during the administration of the painful stimulus and not after its removal. Therefore, it remains possible that IPC can reduce pain intensity during withdrawal of a painful stimulus in young, healthy individuals, and should be considered by future research.

In the current study, it is unknown how IPC reduced time under pain to such a large effect (Cohen’s *d* = 1.3) while having no measurable effect on pain intensity. The data revealed that the reduction in total time under pain was mainly driven by a reduction in the time it took for pain to subside after removal from the painful stimulus. Therefore, it is possible that ischemic pain and the degree of opioid release from 5 min of unilateral IPC was insufficient to decrease the intensity of the cold pain; thereby explaining the presence of an effect only when the painful stimulus was removed. Exercise has also shown to be effective in reducing pain intensity (Cruz et al., 2015) via endogenous opioids (Koltyn, 2000; Koltyn et al., 2014), and indeed a certain exercise intensity and duration is needed before an analgesic effect (Naugle et al., 2012). As such, an augmented IPC stimulus (Cook et al., 1997) may be necessary to elicit an adequate response and observe a reduction in pain intensity during the painful stimulus and this may alter the observed relationship between pain sensitivity and exercise performance.

### Limitations

This study has limitations. Repeated control trials were not conducted; thus, within-subject variability and reproducibility of measured variables are not known. Also, it must be recognized that owing to a limited number of potential participants, low observed power was observed in certain statistical analyses; thus, if the observed effect sizes did indeed reflect the true effect sizes, there was insufficient power in these analyses to produce significant results.

## Conclusion

In a group of young, heathy subjects, IPC reduces pain sensitivity during a painful cold stimulus. However, the current study does not support a strong relationship between pain sensitivity modulation and the ergogenic effect of IPC.

## Data Availability Statement

The raw data supporting the conclusions of this article will be made available by the authors, without undue reservation.

## Ethics Statement

The studies involving human participants were reviewed and approved by the University of Guelph’s Human Research Ethics Board. The patients/participants provided their written informed consent to participate in this study.

## Author Contributions

JS and JB contributed to conception and design of the study. JS organized the database, performed the statistical analysis, and wrote the first draft of the manuscript. Both authors contributed to manuscript revision, read, and approved the submitted version.

## Conflict of Interest

The authors declare that the research was conducted in the absence of any commercial or financial relationships that could be construed as a potential conflict of interest.
